# The complete mitochondrial genome of the intertidal spider (*Desis jiaxiangi*) provides novel insights into the adaptive evolution of the mitogenome and the evolution of spiders

**DOI:** 10.1186/s12862-021-01803-y

**Published:** 2021-04-30

**Authors:** Fan Li, Yunyun Lv, Zhengyong Wen, Chao Bian, Xinhui Zhang, Shengtao Guo, Qiong Shi, Daiqin Li

**Affiliations:** 1 College of Life Sciences, University of Chinese Academy of Sciences, Beijing, 100049 China; 2 Shenzhen Key Lab of Marine Genomics, Guangdong Provincial Key Lab of Molecular Breeding in Marine Economic Animals, BGI Academy of Marine Sciences, BGI Marine, BGI, Shenzhen, 518083 China; 3 Centre for Behavioural Ecology and Evolution, School of Life Sciences, Hubei University, Wuhan, 430062 Hubei China; 4 Key Laboratory of Sichuan Province for Fishes Conservation and Utilization in the Upper Reaches of the Yangtze River, College of Life Sciences, Neijiang Normal University, Neijiang, 641100 China; 5 Department of Biological Sciences, National University of Singapore, Singapore, 117543 Singapore

**Keywords:** Mitogenome, Phylogeny, Evolution, Positive selection, *Desis jiaxiangi*, *Argyroneta aquatica*

## Abstract

**Background:**

Although almost all extant spider species live in terrestrial environments, a few species live fully submerged in freshwater or seawater. The intertidal spiders (genus *Desis*) built silk nests within coral crevices can survive submerged in high tides. The diving bell spider, *Argyroneta aquatica*, resides in a similar dynamic environment but exclusively in freshwater. Given the pivotal role played by mitochondria in supplying most energy for physiological activity via oxidative phosphorylation and the environment, herein we sequenced the complete mitogenome of *Desis jiaxiangi* to investigate the adaptive evolution of the aquatic spider mitogenomes and the evolution of spiders.

**Results:**

We assembled a complete mitogenome of the intertidal spider *Desis jiaxiangi* and performed comparative mitochondrial analyses of data set comprising of *Desis jiaxiangi* and other 45 previously published spider mitogenome sequences, including that of *Argyroneta aquatica*. We found a unique transposition of *trnL2* and *trnN* genes in *Desis jiaxiangi*. Our robust phylogenetic topology clearly deciphered the evolutionary relationships between *Desis jiaxiangi* and *Argyroneta aquatica* as well as other spiders. We dated the divergence of *Desis jiaxiangi* and *Argyroneta aquatica* to the late Cretaceous at ~ 98 Ma. Our selection analyses detected a positive selection signal in the *nd4* gene of the aquatic branch comprising both *Desis jiaxiangi* and *Argyroneta aquatica*. Surprisingly, *Pirata subpiraticus*, *Hypochilus thorelli*, and *Argyroneta aquatica* each had a higher *Ka/Ks* value in the 13 PCGs dataset among 46 taxa with complete mitogenomes, and these three species also showed positive selection signal in the *nd6* gene.

**Conclusions:**

Our finding of the unique transposition of *trnL2* and *trnN* genes indicates that these genes may have experienced rearrangements in the history of intertidal spider evolution. The positive selection signals in the *nd4* and *nd6* genes might enable a better understanding of the spider metabolic adaptations in relation to different environments. Our construction of a novel mitogenome for the intertidal spider thus sheds light on the evolutionary history of spiders and their mitogenomes.

**Supplementary Information:**

The online version contains supplementary material available at 10.1186/s12862-021-01803-y.

## Background

Mitochondria contain unique genome material that is widely used in phylogenetic reconstruction, population genetics, and evolutionary studies to answer many important biological questions [1–8]. Understanding the forces that drive the evolution of the mitochondrial genome (mitogenome) provides crucial information, as this evolution is affected by a range of factors that in turn influence the information content of the genome. The mitogenome of metazoans is a circular double-stranded DNA comprising 37 generally conserved genes, including 13 protein-coding genes (PCGs), 22 transfer RNAs (tRNAs), and 2 ribosomal RNAs (rRNAs; 16S and 12S), which are essential for various mitochondrial functions [9]. Each mitochondrion has its own systems for replication, transcription, and translation. Proteins encoded by the 13 PCGs are related to oxidative phosphorylation, a critical process in producing ATP (adenosine triphosphate) to maintain the energy supply of cells using oxygen and simple sugars [10]. As of April 2020, there were records for 88,254 complete mitogenomes for animals in the NCBI database (https://www.ncbi.nlm.nih.gov/nuccore/?term=mitochondrion+complete+genome). Of them, 72,151 records were for 6129 vertebrate species and 10,559 records were for 5428 arthropod (invertebrate) species. Compared to vertebrates, however, studies on invertebrate mitogenomes are relatively limited [6].

Spiders are highly diverse and play an important role in various ecosystems. With about 50,000 known species [11], spiders form a distinctive, megadiverse, and ancient lineage (> 380 Ma) of predators almost omnipresent in terrestrial ecosystems [12–14]. It is estimated that the global spider community can consume 400–800 tons of prey every year [15]. Spiders are also well known for their production and use of silk. Despite their highly diverse and ecologically important lineage, however, only 45 complete mitogenomes have been reported in the NCBI database (as of April 2020; for more details, see Additional file 1: Table S1).

Moreover, although almost all spiders are terrestrial, a few live fully submerged in water. The diving bell spider, *Argyroneta aquatica* (Clerck, 1757), the sole species of the genus *Argyroneta* Latreille, 1804 (family Dictynidae), is the most representative spider species associated with freshwater, with a fully underwater residence and a publicly available mitogenome [2]. In addition, members of the spider genus *Desis* Walckenaer, 1837 (family Desidae) live fully submerged in seawater. *Desis* spiders inhabit intertidal zones at the junction of sea and land. Although they have a fascinating habitat [16, 17] and have been subject to taxonomic study [18, 19], there is limited molecular research on this lineage [20]. *Desis* spiders are mainly found along the coast of warmer seas in Australia, Brazil, China, Galapagos, India, Japan, New Zealand, Polynesia, and South Africa [19]. They build silken nests under shells, within rock or coral crevices to keep out seawater. During high tides, *Desis* spiders are able to survive in their submerged silken retreats with little oxygen for up to 19 days [16, 18] or even up to 23 days (unpublished data). This unusual biological characteristic makes *Desis* spiders amazing. Their ecological habitat, intertidal zones, is one of the most stressful environments on earth, with dynamic changes in salinity, pH, temperature, and oxygen concentrations [21]. Mitochondria play an important role in aerobic respiration through oxidative phosphorylation [22]. However, no mitogenome assembly has been reported for the family Desidae in general or for the genus *Desis* specifically (as of December 2020). Such studies have the potential to shed light on the molecular mechanisms behind spiders’ adaptation to harsh aquatic environments and their evolution.

Given the critical role played by mitochondria in aerobic respiration and the environment of aquatic spiders, here we explore the adaptive evolution of spider mitogenomes and phylogenetic relationships in spiders. Taking advantage of new sequencing technologies and rapid development in bioinformatics, we sequenced and assembled the complete mitogenome of *Desis jiaxiangi* Lin, Li & Chen, 2020 from Hainan Island, China [23, 24]. To explore the evolution of aquatic spiders, we combined data from all 45 publicly available spider mitogenomes, including that of the fully freshwater *Argyroneta aquatica*. We analyzed this large data set to infer the evolutionary relationships and divergence times of spiders. Using the dated phylogenetic tree, we performed selection analyses on each PCG across the 46 spider species, with a focus on the clade containing terrestrial and aquatic (*Desis jiaxiangi* and *Argyroneta aquatica*) lineages, to detect potential positive selection acting on this aquatic clade. Our study provides new mitogenomic resources and enhances understanding of the molecular mechanisms behind adaptation to the aquatic environment among intertidal spiders and the evolution of spiders.

## Results

### Summary of the mitogenome assembly and annotation

**Fig. 1:**
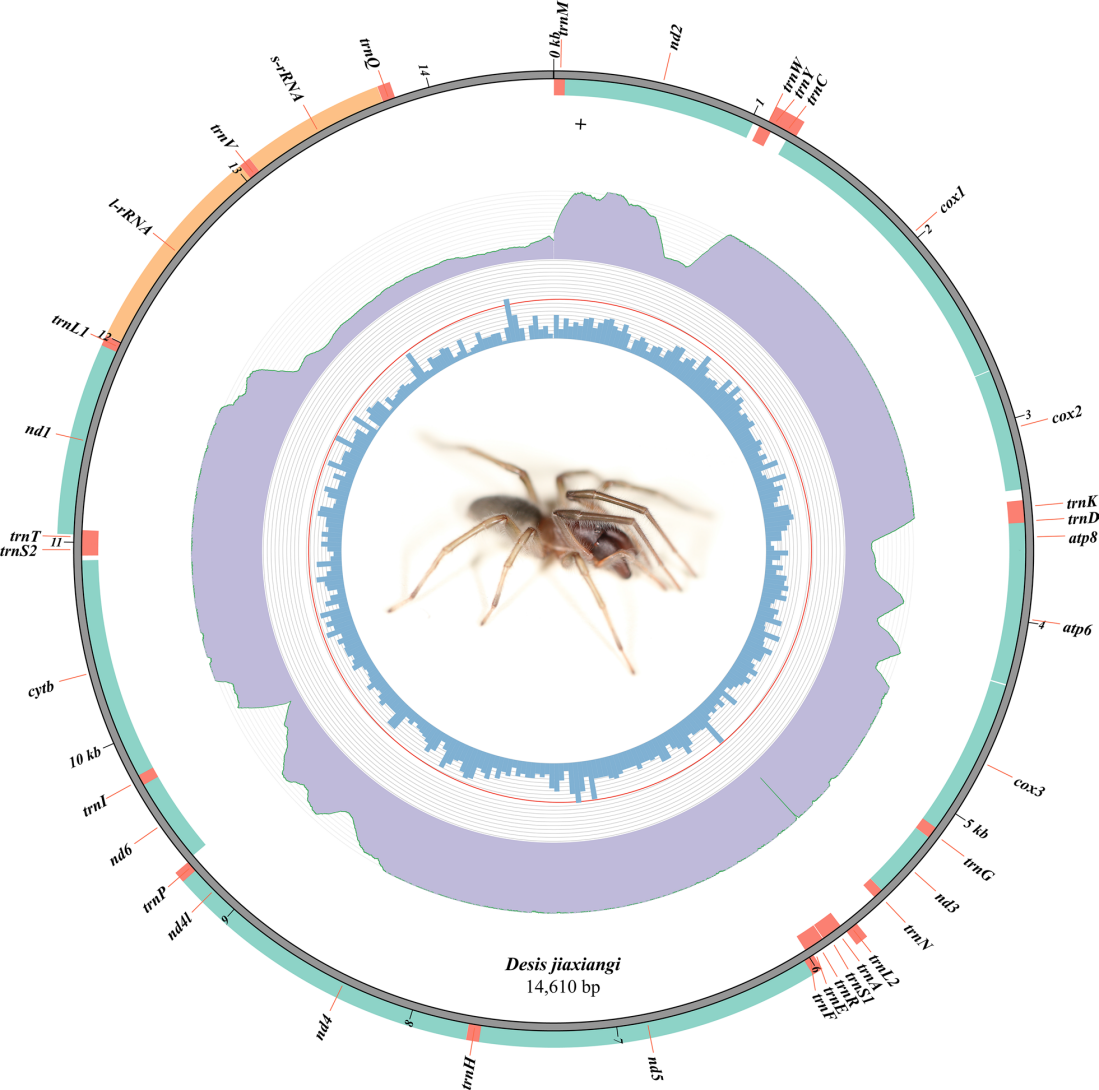
The complete mitogenome of *Desis jiaxiangi*. The innermost and middle circles represent GC content and depth distribution, respectively. The outermost circle shows gene arrangements, with orange fragments for rRNAs, red for tRNAs, and green for PCGs

The primary mitogenome of *Desis jiaxiangi* (see Additional file 2: Fig. S1) assembled by MitoZ was about 14,600 bp (Fig. 1). To validate the assembled sequence, we manually revised the sequence with the mitogenomes of four close species (*Agelena silvatica* Oliger, 1983, *Argyroneta aquatica*, *Telamonia vlijmi* Prószyński, 1984, and *Dolomedes angustivirgatus* Kishida, 1936) using BLAST. After detailed comparisons, we successfully amplified uncertain regions by PCR with the two pairs of designed primers. We then successfully assembled and annotated the mitogenome of *Desis jiaxiangi*.

The final complete mitogenome of *Desis jiaxiangi* (GenBank accession no. MW178198) was 14,610 bp, including 13 PCGs, 22 tRNA genes, 2 rRNA genes, and a noncoding region (D-loop). The majority of genes were encoded on the heavy strand; few genes were encoded on the light strand (for more details, see Table 1 and Fig. 1). The GC content of the entire mitogenome was 23%, and the length of the tRNAs ranged from 49 to 99 bp (Table 1).

**Table 1:** Annotation of the complete mitogenome of *Desis jiaxiangi*

Gene	From	To	Spacer(+)/Overlap(−)	Length(bp)	Start	Stop	Strand
*tRNA*^*Met*^	1	67	0	67			H
*nd2*	55	1014	− 13	960	ATA	TAG	H
*tRNA*^*Trp*^	1048	1111	33	64			H
*tRNA*^*Tyr*^	1078	1146	− 34	69			L
*tRNA*^*Cys*^	1132	1230	− 15	99			L
*cox1*	1198	2739	− 33	1542	GTA	TAA	H
*cox2*	2744	3406	4	663	TTG	TAA	H
*tRNA*^*Lys*^	3403	3469	− 4	67			H
*tRNA*^*Asp*^	3452	3516	− 18	65			H
*atp8*	3514	3657	− 3	144	ATA	TAG	H
*atp6*	3651	4319	− 7	669	ATG	TAA	H
*cox3*	4326	5111	6	786	TTG	TAG	H
*tRNA*^*Gly*^	5111	5170	− 1	60			H
*nd3*	5171	5518	0	348	ATT	TAA	H
*tRNA*^*Asn*^	5519	5568	0	50			H
*tRNA*^*Leu(UUR)*^	5719	5781	150	63			L
*tRNA*^*Ala*^	5787	5847	5	61			H
*tRNA*^*Ser(AGN)*^	5844	5892	− 4	49			H
*tRNA*^*Arg*^	5896	5969	3	74			H
*tRNA*^*Glu*^	5939	5998	− 31	60			H
*tRNA*^*Phe*^	5978	6031	− 21	54			L
*nd5*	6030	7670	− 2	1641	ATT	TAA	L
*tRNA*^*His*^	7658	7719	− 13	62			L
*nd4*	7720	8998	0	1,279	TTG	T	L
*nd4l*	8952	9269	− 47	318	ATT	TAA	L
*tRNA*^*Pro*^	9265	9319	− 5	55			L
*nd6*	9329	9754	9	426	ATA	TAG	H
*tRNA*^*Ile*^	9753	9814	− 2	62			H
*cytb*	9804	10,911	− 11	1,108	ATT	T	H
*tRNA*^*Ser(UCN)*^	10,935	10,993	23	59			H
*tRNA*^*Thr*^	10,993	11,060	− 1	68			H
*nd1*	11,038	11,946	− 23	909	ATT	TAG	L
*tRNA*^*Leu(CUN)*^	11,951	12,003	4	53			L
*16S rRNA*	12,005	13,022	1	1,018			L
*tRNA*^*Val*^	13,023	13,077	0	55			L
*12S rRNA*	13,078	13,765	0	688			L
*tRNA*^*Gln*^	13,767	13,828	1	62			L
D-loop	13,829	14,610	0	782			-

^1^H and L refer to the heavy (majority) and light (minority) strand, respectively

In the mitogenome of *Desis jiaxiangi*, the initiation codon of most PCGs was ATT (5), ATA (3), TTG (3) or ATG (1); the *cox1* was initiated with non-canonical codon (see Table 1). Conversely, the termination codons were TAA (6), TAG (5), T (2).

### Mitochondrial gene rearrangement

**Fig. 2:**
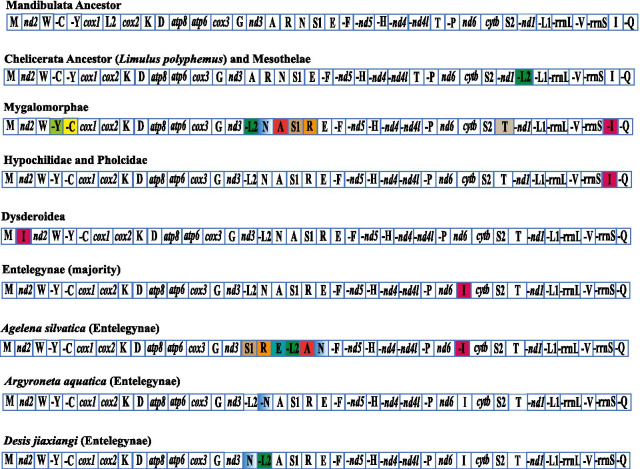
Gene arrangements in spider mitogenomes. The last three species belong to the marronoid clade within the RTA group. The rearranged genes are color-coded, and one color indicates one rearranged gene. “-” refers to genes on the light (minority) strand

We revealed gene rearrangements from Mandibulata ancestor to Chelicerata ancestor, Mesothelae, RTA (retrolateral tibial apophysis) clade, and other major clades (Fig. 2). Compared to the gene order of Mandibulata ancestor, the *trnL2* of Chelicerata ancestor and Mesothelae changed its position to a new location between *nd1* and *trnL1*, and also moved from heavy to light strand. More inversion and transpositions were observed in Mygalomorphae (Fig. 2). The lineages in Entelegynae have a major gene order (Fig. 2). Within the RTA clade, *Agelena silvatica*, *Argyroneta aquatica*, and *Desis jiaxiangi* are classified as marronoids (Fernandez et al. [25]; Wheeler et al. [12]). The gene orders found in these three marronoid spider mitogenomes were clearly different from one another. The mitogenome structures of the two aquatic spiders, *Argyroneta aquatica* and *Desis jiaxiangi*, were very similar, except for the unique transposition of *trnL2* and *trnN*. In addition, *trnN* was encoded from the light strand instead of the heavy strand in these two aquatic species. However, compared to their terrestrial counterpart *Agelena silvatica*, more significant changes were visible. For example, in the freshwater and intertidal spiders, *trnL2* was inserted after *nd3*; *trnA* and *trnN* were interchanged; *trnS1*, *trnR*, and *trnE* were accordingly located after *trnA*. Unlike the major lineages of Entelegynae that had *trnN* in the light strand, the freshwater spider *Argyroneta aquatica* had *trnN* in the heavy strand. However, the gene orders of all PCGs were conserved within Araneae, given that gene order changes occurred only in tRNA genes (for more details, see Fig. 2).

### Phylogenetic and divergence time analyses

**Fig. 3:**
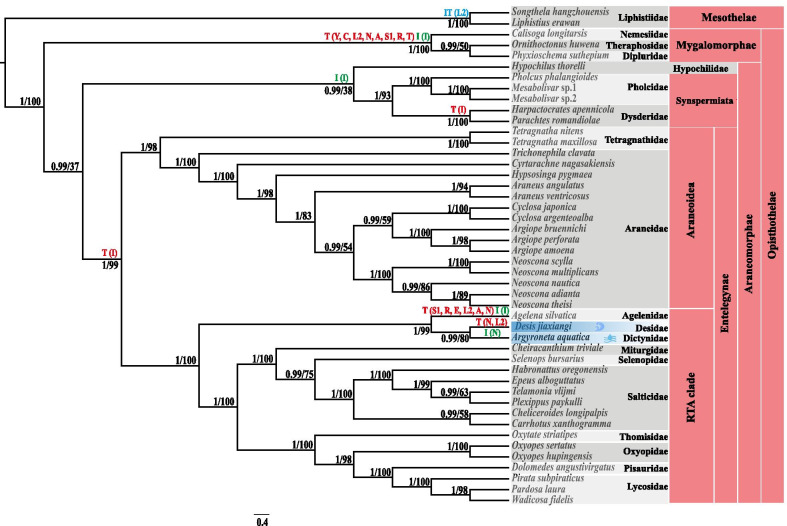
Phylogeny of spiders inferred from the amino acid sequences of 13 PCGs of the 46 spider mitogenomes examined using both Bayesian inference (BI) and maximum likelihood (ML) methods. Values are shown next to nodes, with posterior probability on the left and ML bootstrap support values on the right. Taxa highlighted in blue are in the aquatic branch. The rearranged genes are marked on the branches with T (Transposition), I (Inversion), IT (Inverse transposition)

The BI and ML phylogenetic trees, based on amino acid sequence data from 13 PCGs derived from the 46 spider mitogenomes (see Additional file 1: Table S1), were identical (see Fig. 3). Both the BI and ML trees strongly supported the monophyly of the suborder Mesothelae, the infraorders Mygalomorphae and Araneomorphae. Both the BI and ML trees also strongly supported the lineage containing Agelenidae, Desidae, and Dictynidae, which belonged to the marronoid clade (posterior probability = 1, bootstrap = 99). Meanwhile, the families Desidae and Dictynidae both belonged to the superfamily Dictynoidea.

*Desis jiaxiangi* is sister to *Argyroneta aquatica*, which implies a close relationship between these two lineages. In addition, the clade *Desis jiaxiangi* + *Argyroneta aquatica* is sister to *Agelena silvatica*. Our findings support all of the major backbone lineages within Araneae, recovering a deep split between the two suborders, Mesothelae and Opisthothelae (Mygalomorphae and Araneomorphae). The most diverse Araneomorphae lineage encompasses Hypochilidae, Synspermiata, Araneoidea and RTA clade. These evolutionary relationships are consistent with a previous study based on transcriptome data [25]. Because of the lack of public mitogenome data for the family Filistatidae, the Hypochilidae clade is closed to the clade of Synspermiata. According to previous studies [12, 25], within Araneomorphae, the Hypochilidae + Filistatidae cluster is sister to Synspermiata and constitutes the sister group of all other clades in Araneomorphae. Our findings also support this view (for more details, see Fig. 3).

**Fig. 4:**
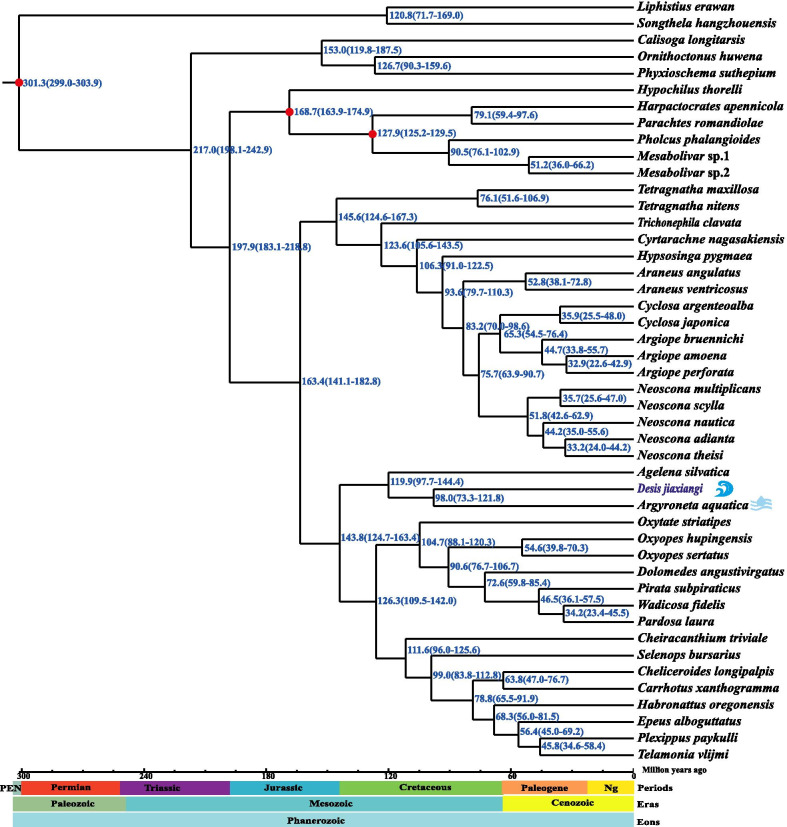
Estimates of divergence time of spiders with three fossil calibration points (red dots) inferred from an analysis of 46 complete mitogenomes using Bayesian MCMCTree with the independent-rates relaxed-clock model. Model = HKY85. Values are shown next to nodes with mean estimates and 95% confidence intervals

Based on the three-fossil-calibrated phylogeny (Fig. 4), we dated the most recent common ancestor (MRCA) of *Desis jiaxiangi* and *Argyroneta aquatica* to the late Cretaceous at ~ 98 Ma (95% confidence interval: 73–122 Ma).

### Selection analyses

**Fig. 5:**
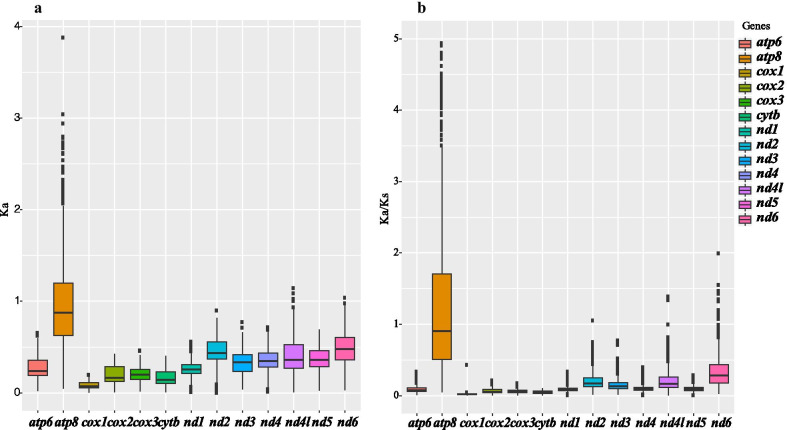
Boxplots of (**a**) *Ka* and (**b**) *Ka/Ks* for the 13 mitochondrial protein-coding genes of the 46 spider mitogenomes examined. The average *Ka* and *Ka/Ks* were greatest in *atp8*

**Fig. 6:**
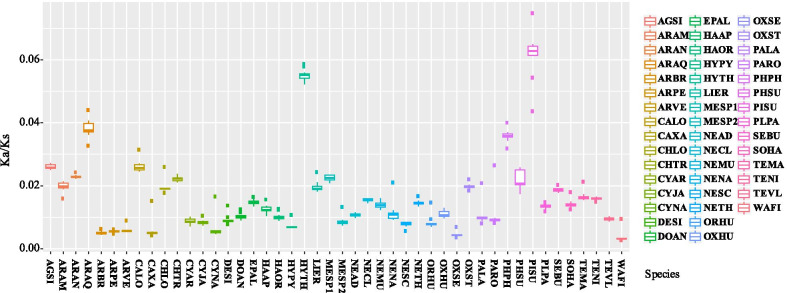
Boxplot of Ka/Ks of the 13 PCGs across the 46 spider mitogenomes examined. These data were calculated with the free-ratios model. Abbreviations correspond to the first two letters of the full species name (see Additional file 1: Table S1). DESI represents *Desis jiaxiangi*

Of all values of *Ka* and *Ka/Ks* calculated from the 13 PCGs of the 46 spider mitogenomes examined, *atp8* had the highest averages of *Ka* and *Ka*/*Ks*. This implies that *atp8* might have evolved more quickly than the other PCGs in the spider mitogenomes (see Fig. 5). Conversely, *cox1* had the lowest averages of *Ka* and *Ka*/*Ks*. When *Ka/Ks* was compared among the different lineages for all 13 PCGs dataset (free-ratios model), all the *Ka/Ks* values are lower than 1. Interestingly, *Pirata subpiraticus*, *Hypochilus thorelli*, and *Argyroneta aquatica* (the freshwater spider) had higher *Ka/Ks* values than other species, whereas the intertidal spider (*Desis jiaxiangi*) had a relatively lower *Ka/Ks* value (Fig. 6).

Furthermore, the results from both the branch and branch-site models showed significant positive selection on the ancestral branch of *Desis jiaxiangi* and *Argyroneta aquatica* with *nd4* (*p* < 0.05; Table 2). There was no significant positive selection signal from both the branch and branch-site models when testing *Pirata subpiraticus*, *Hypochilus thorelli*, *Argyroneta aquatica*, *Desis jiaxiangi* and *Agelena silvatica* as the foreground independently (Tables 3, 4, 5, 6, 7). Nevertheless, the *nd6* showed significant positive selection in the branch-site model in the three species with the highest *Ka/Ks* value (i.e., *Pirata subpiraticus*, *Hypochilus thorelli*, *Argyroneta aquatica*) when tested independently (*p* < 0.05; Tables 3, 4, 5).

**Table 2:** Chi-square tests based on the branch model and branch-site model analyses when testing the clade comprising both *Desis jiaxiangi* and *Argyroneta aquatica* as the foreground

Gene	*p*-value in branch model	*p*-value in branch-site model
*atp6*	0.239	0.072
*atp8*	NA	1.000
*cox1*	**0.006**	0.061
*cox2*	0.386	0.991
*cox3*	**0.028**	0.365
*cytb*	**0.034**	0.739
*nd1*	0.260	0.277
*nd2*	0.538	0.587
*nd3*	0.960	1.000
***nd4***	**0.020**	**0.017**
*nd4l*	0.745	0.598
*nd5*	0.269	0.415
*nd6*	0.618	0.243

NA: not applicable. A significant positive selection is in bold

**Table 3:** Chi-square tests based on the branch (*Pirata subpiraticus*) model and branch-site model analyses

Gene	*p*-value in branch model	*p*-value in branch-site model
*atp6*	0.589	1.000
*atp8*	NA	1.000
*cox1*	0.551	** < 0.0001**
*cox2*	0.205	1.000
*cox3*	0.971	0.212
*cytb*	0.288	**0.047**
*nd1*	0.933	0.991
*nd2*	0.128	0.811
*nd3*	**0.019**	0.411
*nd4*	**0.005**	0.193
*nd4l*	0.791	1.000
*nd5*	0.285	0.615
*nd6*	0.543	**0.009**

NA, not applicable. A significant positive selection is in bold

**Table 4:** Chi-square tests based on the branch (*Hypochilus thorelli*) model and branch-site model analyses

Gene	*p*-value in branch model	*p*-value in branch-site model
*atp6*	0.055	1.000
*atp8*	NA	0.419
*cox1*	0.089	1.000
*cox2*	0.704	0.999
*cox3*	0.766	0.150
*cytb*	0.651	1.000
*nd1*	0.658	0.500
*nd2*	0.801	1.000
*nd3*	0.640	0.298
*nd4*	0.741	1.000
*nd4l*	0.161	0.640
*nd5*	0.151	1.000
*nd6*	0.708	**0.009**

NA, not applicable. A significant positive selection is in bold

**Table 5:** Chi-square tests based on the branch (*Argyroneta aquatica*) model and branch-site model analyses

Gene	*p*-value in branch model	*p*-value in branch-site model
*atp6*	0.778	0.124
*atp8*	NA	1.000
*cox1*	0.990	0.941
*cox2*	0.948	0.933
*cox3*	**0.018**	0.696
*cytb*	**0.008**	0.078
*nd1*	0.097	0.420
*nd2*	0.399	1.000
*nd3*	0.551	1.000
*nd4*	0.553	0.066
*nd4l*	0.804	0.351
*nd5*	0.716	1.000
*nd6*	0.252	**0.004**

NA, not applicable. A significant positive selection is in bold

**Table 6:** Chi-square tests based on the branch (*Desis jiaxiangi*) model and branch-site model analyses

Gene	*p*-value in branch model	*p*-value in branch-site model
*atp6*	0.081	0.331
*atp8*	NA	**0.009**
*cox1*	0.335	0.206
*cox2*	**0.001**	0.541
*cox3*	0.676	**0.038**
*cytb*	**0.032**	0.518
*nd1*	0.278	0.786
*nd2*	0.609	0.824
*nd3*	**0.022**	0.999
*nd4*	0.941	1.000
*nd4l*	0.968	0.993
*nd5*	0.230	1.000
*nd6*	0.057	0.702

NA, not applicable. A significant positive selection is in bold

**Table 7:** Chi-square tests based on the branch (*Agelena silvatica*) model and branch-site model analyses

Gene	*p*-value in branch model	*p*-value in branch-site model
*atp6*	0.299	0.480
*atp8*	NA	**0.034**
*cox1*	0.457	1.000
*cox2*	0.056	0.171
*cox3*	0.079	0.631
*cytb*	0.489	0.129
*nd1*	0.445	1.000
*nd2*	0.979	0.865
*nd3*	0.909	1.000
*nd4*	**0.041**	1.000
*nd4l*	0.557	1.000
*nd5*	0.084	1.000
*nd6*	0.359	0.983

NA, not applicable. A significant positive selection is in bold

## Discussion

The complete mitogenome of *Desis jiaxiangi* is comparable in size to those of other spiders. There is no obvious mitogenome expansion or contraction within available spider mitogenomes during the diversification process. Although mitogenome rearrangements are very common in spider lineages [26] and also in other arthropods, such as crabs [27] and beetles [28], the unique tRNA gene rearrangements were detected in the mitogenomes of the two aquatic spider species, *Argyroneta aquatica* (family: Dictynidae) and *Desis jiaxiangi* (family: Desidae). Compared to spider lineages in the clade Entelegynae, the translocation of *trnL2* and *trnN* was identified in *Desis jiaxiangi*, and *trnN* changed the linked strand in *Argyroneta aquatica*. More novel mitogenomes from these two families (Desidae and Dictynidae), to which *Argyroneta aquatica* and *Desis jiaxiangi* belong, respectively, are thus needed to determine whether these gene arrangements are the shared gene order pattern within each of these two spider families.

These two species within the superfamily Dictynoidea can be used as good models for comparative genome studies, because they occupy completely different ecological environments. *Argyroneta aquatica* lives almost entirely in freshwater, whereas *Desis jiaxiangi* resides in intertidal zones and can survive long periods submerged in seawater during high tides. *Desis* spiders are so-called semiaquatic spiders, as they hunt in intertidal zones during low tides like terrestrial spiders [29]. Therefore, *Desis* spiders must adapt to both aquatic and terrestrial conditions. *Desis formidabilis* was confirmed to have high hemolymph, the spider’s blood, in concentrations similar to those of marine crustaceans [30]. This feature is very well adapted to intertidal zones. The hydrofuge hairs on the bodies of aquatic spiders can form a thin air film, which enables them to use a physical gill or plastron respiration to exchange oxygen and carbohydrates [29, 31]. Meanwhile, *Desis marina* has lower respiration rates than other spider species [17]. In general, many organisms highly diverged in the opposite osmotic pressures induced by freshwater or seawater. While these two aquatic genera (*Argyroneta* and *Desis*) occupy different habitats, they have similar strategies and morphological features to prevent water from entering the book lungs and tracheae [32, 33].

It is noted that the terrestrial counterpart, *Agelena silvatica*, has many more gene rearrangements than the aquatic spiders. As the majority of terrestrial spiders in Entelegynae share the similar gene order (see Fig. 2), the significant gene transpositions in *Agelena silvatica* are uncertain. Further mitogenome sequencing of spiders in the marronoid clade is thus required.

The molecular dating analysis estimated that the MRCA of *Desis* and *Argyroneta* spiders diverged around 98 Ma, which is consistent with the extreme age (> 90 Myr) of major adaptations of spiders with aquatic lifestyles (superfamily Dictynoidea) [32]. This result presents a helpful timeline for clarifying the evolution of aquatic spiders.

Our phylogenetic analyses provided a robust phylogeny for spiders. Most tree nodes with high support values (bootstrap = 100, posterior probability = 1) reconstructed the family relationships, such as Liphistiidae, Tetragnathidae, and Araneidae. The sister relationships are also revealed by high support values, for example, between Desidae and Dictynidae (bootstrap = 80, posterior probability = 0.99). Therefore, PCGs (amino acid sequences) in mitochondria can be considered reliable molecular markers for phylogenetic analyses of various spiders.

Without a doubt, all 13 PCGs in the mitochondria of living creatures are important to their aerobic metabolism. Positive selection may provide important functional details associated with adaptation to new environments [5]. For example, positive selection in *nd4*, *cytb*, and *atp8* is assumed to have played a critical role in the origin of flight in bats to meet the huge change in energy demand [34]. Positive selection in *atp6*, *nd2*, and *nd4* has been found for galliforms’ adaptation to high altitudes [22]. Our selective analyses revealed significant positive selection signals in *nd4* on the branch of two aquatic spider lineages, *Argyroneta aquatica* and *Desis jiaxiangi*. In the spider mites (*Tetranychus truncates* and *Tetranychus pueraricola*), a positive selection on *nd4* and *atp6* was also detected and assumed to be associated with the different climate adaptations [35]. The peptides encoded by *nd4* constitute mitochondrial complexes I, which participate in oxidative phosphorylation (OXPHOS); the amino acid changes within *nd4* also affect the efficiency of ATP synthesis [35]. Like the high-altitude environments of galliforms, aquatic environments are short of oxygen, which implies association between gene positive selection and adaptation to energy metabolism in aquatic environments [22, 36]. While our study did not directly test the adaptation to aquatic environment, our results can inspire further investigation of aquatic adaptations of spider evolution.

The *atp8* gene with the highest averages of both *Ka* and *Ka*/*Ks* suggests more changes in amino acids. This is also consistent with the previously identified *Ka/Ks* ratios in other spiders [37], insects [36] and fishes [38]. The higher *Ka*/*Ks* values indicate that the *atp8* gene might have experienced more relaxed selective constraints and accumulated more mutations, thus it would be more likely to lose its function [36]. The *cox1* gene with lowest *Ka* and *Ka*/*Ks* value suggests that it might have experienced more strong evolutionary pressures [36]. Therefore, *cox1* has been widely used as barcoding marker for reconstructing phylogenies of spiders and other taxa. In the free-ratios model, *Ka*/*Ks* values for all 13 PCGs are less than 1 (Fig. 6), suggesting that purifying selection may have predominated the evolution of mitogenomes, as shown in spider mites [35] and insects [36].

Surprisingly, the much higher *Ka/Ks* values were detected in *Pirata subpiraticus*, *Hypochilus thorelli* and *Argyroneta aquatica*. This finding indicates that these three species may have higher nonsynonymous substitution rates than other examined species in this study. The higher nonsynonymous mutations suggest that these species may have experienced more relaxed evolutionary constraints [36] and might have fixed more slightly beneficial amino acid changes [22]. When testing these three species and the intertidal species as the foreground independently, the *nd4* gene had no significant signal from both the branch and branch-site models. This may be because the aquatic spider species (*Argyroneta aquatica* or *Desis jiaxiangi*) existed in the background branches when analysed. In other words, a significant positive selection signal in *nd4* can be revealed only when the foreground branch contained both *Desis jiaxiangi* and *Argyroneta aquatica*. However, the *nd6* gene were detected to have significant signal in the branch-site models, but not from both the branch and branch site models which demonstrate a strong positive selection, in the three species, *Pirata subpiraticus*, *Hypochilus thorelli* and *Argyroneta aquatica* (*p* < 0.05; Tables 3, 4, 5). The *nd6* gene may play an essential role in each evolutionary process of these three species with the highest *Ka/Ks* values. The peptides encoded by *nd6* involve in catalytic synthesis of ATP, and changes within *atp6* have been shown to link to the differences in metabolism [35]. Among these three spiders, *Pirata subpiraticus* and *Argyroneta aquatica* are in the RTA clade, while *Hypochilus thorelli* is in the Hypochilidae clade. *Pirata subpiraticus* is a pond wolf spider with a relatively large body shape and a quick predation ability in paddy field [39]. *Hypochilus thorelli* is considered to be the most primitive araneomorph spider species that builds the lampshade-shaped web. Its preferred habitat is close to a stream and well-shaded ledges [40]. The positive selection signals on *nd6* found in these three species may be relevant to their adaptations on energetic requirements. However, further studies are needed to test this hypothesis.

Mitochondria, as the powerhouses of the cell may also be related to the foraging strategies of spiders. Web-building spiders are ambushers that use their webs as traps to catch prey, whereas hunting spiders have to actively search for prey [41]. Hunting spiders may thus require more energy to find prey than ambushing spiders. Neither *Argyroneta aquatica* nor *Desis jiaxiangi* use webs for foraging; instead, both have to run or swim quickly in search of prey. These spiders also stay under water for long periods in oxygen-limited environments [16, 32]. Thus, these aquatic spiders should be very efficient at metabolizing energy to meet the demands of energy consumption. Positive selection on spider mitochondrial PCGs may have shaped the evolution of aquatic spider lineages.

Similarly, recent research on intertidal chitons has also detected positive selection on their mitochondrial PCGs, which may help the chitons adapt to contrasting environments [21]. Although our analyses revealed significant positive selection signals in *nd4* on the branch of two spider species, *Argyroneta aquatica* and *Desis jiaxiangi*, our data set did not cover all spider lineages; hence, more studies of the mitogenomes of Desidae, Dictynidae, and other families are required. Furthermore, the higher *Ka/Ks* values and the positive selection on the *nd6* gene in *Pirata subpiraticus*, *Hypochilus thorelli* and *Argyroneta aquatica* warrant for more studies. Because of limited data, comparative genome studies on spider lineages remain largely underexploited. It is hoped that our study stimulates more genome studies on spiders, especially aquatic spiders, with the expectation of revealing more details on the molecular mechanisms behind the adaptation of intertidal spiders to marine environments.

## Conclusions

The fact that *Desis* spiders can live under seawater at high tides shows how well they have adapted to extremely harsh conditions and how they are able to tolerate limited oxygen and seawater salinity. Here we provide a complete mitogenome sequence of spiders from the family Desidae. The different mitogenome orders of *Argyroneta aquatica* and *Desis jiaxiangi* imply they have undergone a different divergent evolution in the gene order of their mitogenome. Both BI and ML phylogenetic analyses supported the close relationship between *Argyroneta aquatica* and *Desis jiaxiangi*, and our dating analyses revealed that they diverged in the late Cretaceous. The positive selection acting on *nd4* on the branch consisting of both *Argyroneta aquatica* and *Desis jiaxiangi* may affect the efficiency of their ATP synthesis. The three species, *Pirata subpiraticus*, *Hypochilus thorelli* and *Argyroneta aquatica,* have higher *Ka/Ks* values than other species in the 13 PCGs dataset and have been detected positive selection signals on *nd6*. This interesting finding may be relevant to their adaptations on energetic requirements. More in-depth analysis on these clades could build up our knowledge towards their metabolic adaptations. In summary, our study probably provides molecular evidence of the evolution of an aquatic lifestyle and presents a new genetic resource for phylogenetic studies in spiders.

## Methods

### Sample collection

In December 2018, we visited intertidal zones in Sanya City, Hainan Province, China. During low tides, we carefully searched coral crevices in the intertidal zone and collected 13 specimens of *Desis jiaxiangi* (see Additional file 2: Fig. S1) in the location (N18.21999°, E109.51128°, 3 m a.s.l.). Voucher specimens were deposited in the Marine Bank of the China National GeneBank (CNGB voucher nos. Des_001 to Des_013), Shenzhen, China.

### Assembly and annotation of the complete mitogenome of *Desis jiaxiangi*

We extracted total DNA from the whole body of one specimen (Des_002) for traditional whole-genome shotgun sequencing using a Puregene Tissue Core Kit A (Qiagen, Germantown, MD, USA). We constructed the library with a short-insert size of 500 bp using standard protocols (Illumina, San Diego, CA, USA). We sequenced with paired-end reads (150 bp in length) on an Illumina Hiseq X Ten platform at BGI-Wuhan, China. The raw sequencing data was filtered by SOAPnuke [42] to trim adapter, low quality, high N base ratio and etc. Finally, we acquired about 100 Gb of clean reads after the sequencing and data filtering. We used all of these clean data as an input file to the Python3-based mitogenome assembly toolkit MitoZ [43] for a primary mitogenome assembly of *Desis jiaxiangi*.

Subsequently, we performed a BLAST search to compare this mitogenome assembly with corresponding mitogenome data of four relatively close spider species from RTA clade: *Agelena silvatica* (GenBank Accession no. KX290739.1), *Argyroneta aquatica* (No. NC_026863.1), *Dolomedes angustivirgatus* (No. NC_031355.1), and *Telamonia vlijmi* (No. KJ598073.1). We manually annotated the conserved regions of our assembled *Desis* mitogenome based on the other four mitogenomes. For the uncertain regions of the assembled *Desis* mitogenome, we designed two pairs of primers (see Additional file 1: Table S2) using Primer Premier 5 (Premier Biosoft, Palo Alto, CA, USA) to obtain the missing sequences using PCR. To validate the sequences of the uncertain regions, we extracted genomic DNA from the leg tissue of the specimens using a Puregene Tissue Core Kit A (Qiagen).

We performed PCR in 50 µL volume tubes, each containing 25 µL 2 × Taq PCR MasterMix (Tiangen Biotech, Beijing, China), 2 µL of each primer (10 µM), and 2 µL genomic DNA (100 ng/µL), with 19 µL double-distilled water. The PCR protocol was as follows: pre-denaturation at 94 °C for 5 min, 35 cycles of denaturation at 94 °C for 30 s, annealing at 48 °C for 30 s, elongation at 72 °C for 1 min 40 s, and a final elongation at 72 °C for 10 min. A Veriti Thermal Cycler (Applied Biosystems, Carlsbad, CA, USA) was used for the PCR. We checked the PCR products by electrophoresis using 1% agarose gels.

After successfully amplifying the uncertain regions, we obtained the final assembly of the complete mitogenome of *Desis jiaxiangi*, which was annotated with MITOS2 [44] on MITOS WebServer (http://mitos2.bioinf.uni-leipzig.de/index.py). The final mitogenome sequence was visualized with MitoZ in the visualize module [43].

Comparative analysis of mitochondrial gene orders is a powerful method of revealing ancient events in the process of species evolution [45]. After completely annotating the mitogenome of *Desis jiaxiangi*, we compared its gene order with the available 45 complete spider mitogenomes (see Additional file 1: Table S1) from the NCBI GenBank.

### Phylogenetic analyses and estimation of divergence time

We prepared a data set comprised the novel mitogenome of *Desis jiaxiangi* and the complete mitogenomes of 45 other spider species (see detailed species names in Additional file 1: Table S1) downloaded from the NCBI GenBank. We performed phylogenetic analyses of this data set using Bayesian inference (BI) and maximum likelihood (ML) methods. We aligned the nucleotide and amino acid sequences of the 13 PCGs from each mitogenome using the multiple sequence alignment program Clustal Omega in EMBL-EBI [46]. We performed a best-fit model selection of amino acid replacement using ProtTest-3.4.2 [47]. Based on Akaike’s information criterion, MtREV + I + G was chosen as the best model for both inference methods. We reconstructed the phylogenetic tree with the ML method using PhyML 3.0 [48]. The node support values were estimated with 100 replicates and other parameters as the default. We also performed BI using MrBayes v3.2.6 [49] to compare the topologies of the ML phylogenetic trees. MCMC algorithm parameters were set for two independent runs with four chains (one cold chain and three heated chains) for 10,000,000 cycles. The sample frequency parameter was set at 1000 for sampling each chain every 1000 cycles. The first 25% of the runs were discarded as burn-in. We used FigTree v1.4.4 (http://tree.bio.ed.ac.uk/software/figtree/) to visualize the derived BI and ML trees.

We used the Bayesian MCMCTree program in PAML package v4.9j [50] to estimate divergence times. Optimized parameters were as follows: clock = independent rates, model = HKY85, nsample = 20,000, burn-in = 2,000. We used calibration points from recommended fossils and a related time in a recent spider fossil review [51]. Given the limited spider species with available mitogenomes, we used three fossils to calibrate the phylogenetic tree: *Palaeothele montceauensis* (299–304 Ma) for the Mesothelae stem, *Eoplectreurys gertschi* (164–175.1 Ma) for the Synspermiata stem, and *Montsecarachne amicorum* (125–129.4 Ma) for the Synspermiata crown. As the fossil of *Almolinus ligula* (43–47.8 Ma) in the Salticidae crown can be placed in the superfamily Hisponinae [51], and no sample mitogenome is available for this superfamily, this fossil was not used for calibration in the present work.

### Selection analyses

To evaluate potential adaptive evolution in the mitochondrial genes of intertidal *Desis* spiders, we performed positive selection analyses using PAML4.9j [50]. Synonymous substitutions in protein-coding sequences cannot cause changes in amino acids, which are typically found in the third, or sometimes first, position of a codon [52]. We thus used a gene-level approach based on the ratio of nonsynonymous (*Ka*) to synonymous (*Ks*) substitution rates to detect potential positive selection signal on PCGs across closely related or divergent species [52, 53]. A *Ka/Ks* ratio of 1, <1, or> 1 in protein-coding sequences may be interpreted as a neutral mutation, a negative (purifying) selection, or a positive (diversifying) selection, respectively [53]. To investigate the variation in nucleotide substitution rates in spider mitogenomes, we retrieved all 13 mitochondrial PCGs (*nd2*, *cox1*, *cox2*, *atp8*, *atp6*, *cox3*, *nd3*, *nd5*, *nd4*, *nd4l*, *nd6*, *cytb*, and *nd1*) from each annotated mitogenome of the 46 spider species examined. We aligned each gene separately with the codon-based model in the Muscle module of MEGA7 [54]. Ambiguous regions in each alignment were removed with Gblocks v0.91b [55]. *Ka*, *Ks*, and the *Ka/Ks* ratio across all 13 PCGs were calculated with KaKs_Calculator v2.0 [53].

To examine positive selection pressure on individual PCGs of the 46 spider species, we generated conserved blocks from codon alignments of each PCG using Gblocks v0.91b. We ran both the branch and branch-site models in the CodeML program of PAML on those codons from the conserved blocks. We used the phylogenetic topology inferred from PhyML as the guide tree for PAML analyses. In branch models, the free *Ka*/*Ks* ratio model is allowed to vary among branches to detect positive selection on the foreground branch. In branch-site models, the one-ratio model assumes that all branches have an identical *Ka*/*Ks* ratio, whereas the two-ratio model assumes that the foreground branch has a different *Ka*/*Ks* ratio from the background branches. The *Ka*/*Ks* ratios in branch-site models are thus allowed to vary both among sites and across branches to detect positive selection on a few sites along the foreground branch. The branch-site model A null fixed all *Ka*/*Ks* ratios to 1, whereas the branch-site model A (positive selection model) did not fix the *Ka*/*Ks* ratio. The branch-site model A was used to detect positive selection sites along the lineages of aquatic spiders (i.e., the foreground branch). The presence of a site with *Ka*/*Ks* ratio > 1 is suggested when the positive selection model A fits the data significantly better than the corresponding model A null.

We first used the free-ratios model (branch model) to calculate the average *Ka/Ks* ratio for all 13 PCGs in each lineage to represent their evolutionary rate of mitogenomes. We paid particular attention to two aquatic species, the intertidal spider (*Desis jiaxiangi*) and the freshwater spider (*Argyroneta aquatica*), which have aquatic habitats, whereas the other species are almost all limited to land. For this purpose, we marked the ancestral branch containing both *Desis jiaxiangi* and *Argyroneta aquatica* as the foreground branch and the rest of the species as the background branches. To compare the results, we marked the branches of *Pirata subpiraticus*, *Hypochilus thorelli*, *Argyroneta aquatica*, *Desis jiaxiangi* and *Agelena silvatica* separately to test these five species independently. We ran the branch models (one-ratio model vs. two-ratio model) and branch-site models (null model A vs. model A) as two pair models using likelihood ratio tests and chi-square tests, respectively, to detect whether the positive selection signals were significant. The null hypothesis assumed that all branches had a common *Ka*/*Ks* ratio. An alternative hypothesis assumed that the ancestral branch had an independent *Ka/Ks* ratio that differed significantly from the background branches with a common *Ka/Ks* ratio. If this hypothesis has a better fit than the null hypothesis, we considered the occurrence of positive selection in the ancestor of aquatic spiders in certain gene(s) when *p* < 0.05. Positive selection does not usually generate the function on the whole length of the target genes, and only a few sites can reflect the positive selection signal. The branch-site models further identified the positive site(s), and the Bayes empirical Bayes analysis was used to calculate posterior probabilities to detect those sites under positive selection.

## Supplementary Information

**Additional file 1: Table S1.** Reported complete mitogenomes from the NCBI database; **Table S2.** Primers used to amplify uncertain fragments.

**Additional file 2: Fig. S1.** Morphology and habitat of the intertidal spider, *Desis jiaxiangi.*

## Data Availability

The final complete mitogenome sequences of *Desis jiaxiangi* in this manuscript are deposited in NCBI with Accession Number MW178198.

## Abbreviations

ATP: Adenosine triphosphate
BI: Bayesian inference
CNGB: China National GeneBank
ML: Maximum likelihood
MRCA: Most recent common ancestor
PCG: Protein-coding gene
rRNA: Ribosomal RNA
RTA: Retrolateral tibial apophysis
tRNA: Transfer RNA
