# Supplementary figures and images for: The complete mitochondrial genome of the intertidal spider (Desis jiaxiangi) provides novel insights into the adaptive evolution of the mitogenome and the evolution of spiders
Source: BMC Ecol Evol. 2021 Apr 30;21:72. doi: 10.1186/s12862-021-01803-y (PMC8086345; doi:10.1186/s12862-021-01803-y)

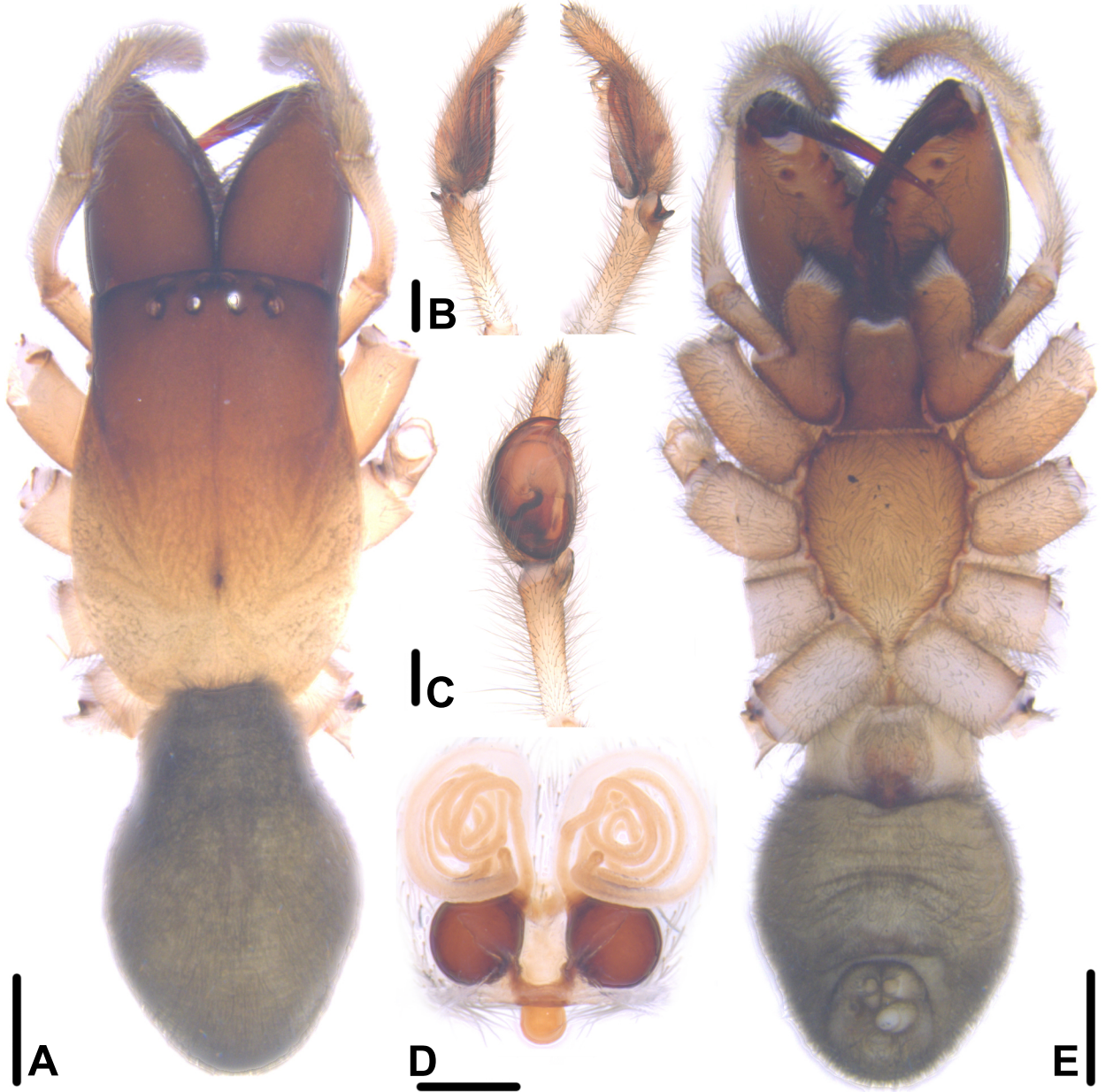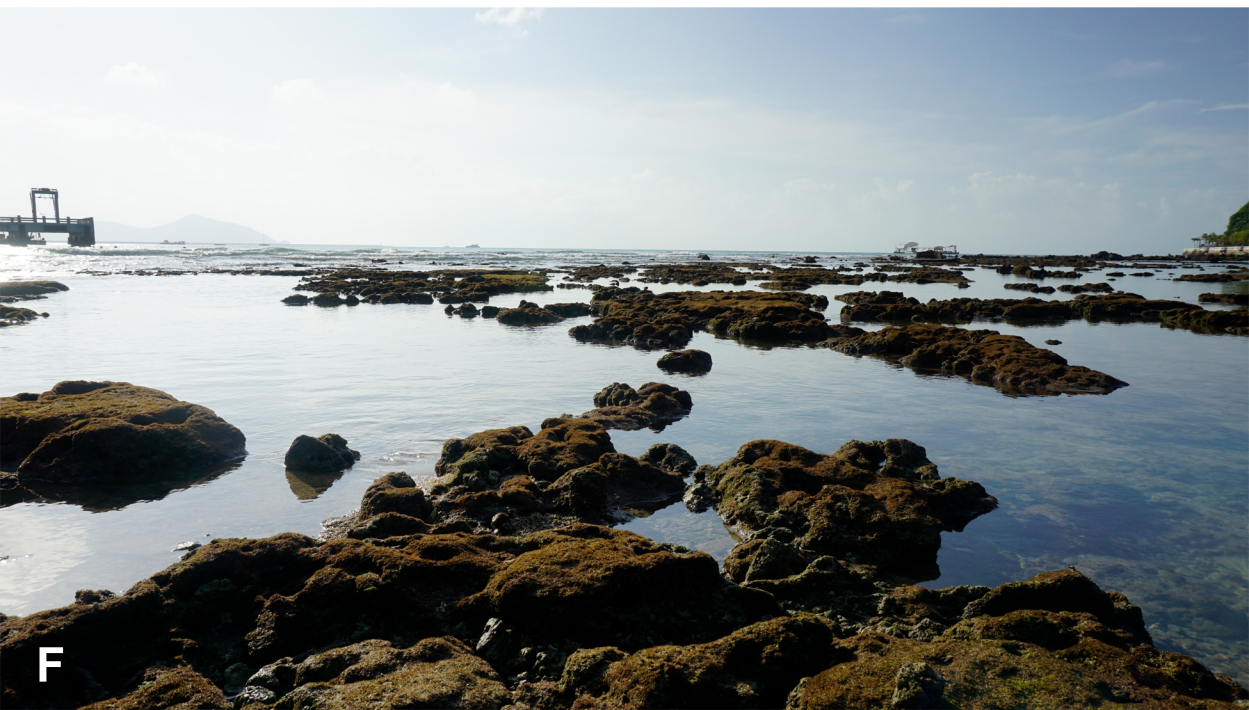

Supplement: Supplementary file 1 — Additional file 1: Table S1. Reported complete mitogenomes from the NCBI database; Table S2. Primers used to amplify uncertain fragments. [file 12862_2021_1803_MOESM1_ESM.pdf]
