# Supplementary material for: The complete mitochondrial genome of the intertidal spider (Desis jiaxiangi) provides novel insights into the adaptive evolution of the mitogenome and the evolution of spiders
Source: BMC Ecol Evol. 2021 Apr 30;21:72. doi: 10.1186/s12862-021-01803-y (PMC8086345; doi:10.1186/s12862-021-01803-y)
Supplement: Supplementary file 2 — Additional file 2: Fig. S1. Morphology and habitat of the intertidal spider, Desis jiaxiangi. [file 12862_2021_1803_MOESM2_ESM.docx]

Appendix A. Supplementary material

**Table S1** Reported complete mitogenomes in the NCBI database.

|  | **Sub(Infa)order** | **Superfamily/Clade** | **Family** | **Species** | **Species abbreviations** | **Accession** | **Length (bp)** | **Submitted Institution** | **Releasing Date** |
| --- | --- | --- | --- | --- | --- | --- | --- | --- | --- |
| 1 | Araneomorphae | Araneoidea | Tetragnathidae | *Tetragnatha nitens* | TENI | NC_028068.1 | 14,639 | China Jiliang University, China | 12-Apr-16 |
| 2 | Araneomorphae | RTA clade | Salticidae | *Habronattus oregonensis* | HAOR | NC_005942.1 | 14,381 | Department of Energy Joint Genome Institute, USA | 01-Feb-10 |
| 3 | Araneomorphae | Araneoidea | Araneidae | *Argiope perforata* | ARPE | NC_044695.1 | 14,032 | Guiyang College, China | 11-Oct-19 |
| 4 | Araneomorphae | Araneoidea | Araneidae | *Neoscona multiplicans* | NEMU | NC_044653.1 | 14,074 | Guiyang College, China | 11-Oct-19 |
| 5 | Araneomorphae | RTA clade | Pisauridae | *Dolomedes angustivirgatus* | DOAN | NC_031355.1 | 14,783 | China Jiliang University, China | 26-Sep-19 |
| 6 | Araneomorphae | RTA clade | Salticidae | *Epeus alboguttatus* | EPAL | NC_042829.1 | 14,625 | Guiyang College, China | 11-Jul-19 |
| 7 | Araneomorphae | RTA clade | Salticidae | *Cheliceroides longipalpis* | CHLO | NC_041120.1 | 14,334 | Guiyang College, China | 10-Apr-19 |
| 8 | Araneomorphae | Araneoidea | Araneidae | *Araneus angulatus* | ARAN | NC_032402.1 | 14,205 | Jiliang University, China | 04-Jan-17 |
| 9 | Araneomorphae | Araneoidea | Araneidae | *Neoscona theisi* | NETH | NC_026290.1 | 14,156 | Jiliang University, China | 04-Feb-15 |
| 10 | Araneomorphae | RTA clade | Lycosidae | *Wadicosa fidelis* | WAFI | NC_026123.1 | 14,741 | Jiliang University, China | 04-Feb-15 |
| 11 | Araneomorphae | Araneoidea | Tetragnathidae | *Tetragnatha maxillosa* voucher 2013-tet-max-1 | TEMA | NC_025775.1 | 14,414 | Anhui Normal University, China | 18-Dec-14 |
| 12 | Araneomorphae | Araneoidea | Araneidae | *Araneus ventricosus* | ARVE | NC_025634.1 | 14,617 | China Jiliang University, China | 15-Dec-14 |
| 13 | Araneomorphae | RTA clade | Thomisidae | *Oxytate striatipes* | OXST | NC_025557.1 | 14,407 | Kangwon National University, Republic of Korea | 15-Dec-14 |
| 14 | Araneomorphae | RTA clade | Lycosidae | *Pirata subpiraticus* | PISU | NC_025523.1 | 14,528 | China Jiliang University, China | 15-Dec-14 |
| 15 | Araneomorphae | RTA clade | Oxyopidae | *Oxyopes sertatus* voucher 2013-phc-nj-ox-se | OXSE | NC_025224.1 | 14,442 | Anhui Normal University, China | 16-Oct-14 |
| 16 | Araneomorphae | RTA clade | Lycosidae | *Pardosa laura* | PALA | NC_025223.1 | 14,513 | Anhui Normal University, China | 16-Oct-14 |
| 17 | Araneomorphae | RTA clade | Selenopidae | *Selenops bursarius* voucher 2014-sel-bur-2 | SEBU | NC_024878.1 | 14,272 | Anhui Normal University, China | 16-Oct-14 |
| 18 | Araneomorphae | RTA clade | Salticidae | *Plexippus paykulli* voucher 2014-ple-pay | PLPA | NC_024877.1 | 14,316 | Anhui Normal University, China | 16-Oct-14 |
| 19 | Araneomorphae | RTA clade | Salticidae | *Telamonia vlijmi* | TEVL | NC_024287.1 | 14,601 | Kangwon National University, Republic of Korea | 02-Jul-14 |
| 20 | Araneomorphae | Araneoidea | Araneidae | *Cyclosa japonica* | CYJA | NC_044696.1 | 14,687 | Guiyang College, China | 11-Oct-19 |
| 21 | Araneomorphae | Araneoidea | Araneidae | *Neoscona scylla* | NESC | NC_044101.1 | 14,092 | Guiyang College, China | 11-Sep-19 |
| 22 | Araneomorphae | RTA clade | Agelenidae | *Agelena silvatica* | AGSI | NC_033971.1 | 14,776 | Southwest University, China | 09-Mar-17 |
| 23 | Araneomorphae | Araneoidea | Araneidae | *Neoscona adianta* | NEAD | NC_029756.1 | 14,161 | China Jiliang University, China | 12-Apr-16 |
| 24 | Araneomorphae | Araneoidea | Araneidae | *Neoscona nautica* | NENA | NC_029755.1 | 14,049 | China Jiliang University, China | 12-Apr-16 |
| 25 | Araneomorphae | Araneoidea | Araneidae | *Hypsosinga pygmaea* | HYPY | NC_028078.1 | 14,193 | China Jiliang University, China | 01-Nov-15 |
| 26 | Araneomorphae | Araneoidea | Araneidae | *Cyrtarachne nagasakiensis* | CYNA | NC_028077.1 | 14,402 | China Jiliang University, China | 01-Nov-15 |
| 27 | Araneomorphae | Araneoidea | Araneidae | *Cyclosa argenteoalba* | CYAR | NC_027682.1 | 14,575 | China Jiliang University, China | 17-Aug-15 |
| 28 | Araneomorphae | RTA clade | Salticidae | *Carrhotus xanthogramma* | CAXA | NC_027492.1 | 14,563 | China Jiliang University, China | 27-Jul-15 |
| 29 | Araneomorphae | RTA clade | Dictynidae | *Argyroneta aquatica* | ARAQ | NC_026863.1 | 16,000 | Southwest University, China | 02-Jun-15 |
| 30 | Araneomorphae | Araneoidea | Araneidae | *Argiope amoena* | ARAM | NC_024282.1 | 14,121 | Anhui Normal University, China | 02-Jul-14 |
| 31 | Araneomorphae | Araneoidea | Araneidae | *Argiope bruennichi* | ARBR | NC_024281.1 | 14,063 | Anhui Normal University, China | 02-Jul-14 |
| 32 | Araneomorphae | Araneoidea | Nephilidae  (Araneidae) | *Nephila clavata (Trichonephila clavata)* | NECL | NC_008063.1 | 14,436 | Kyungpook National University, Republic of Korea | 08-Jun-06 |
| 33 | Araneomorphae | Synspermiata | Pholcidae | *Mesabolivar* sp1*.* ITV1036I2 | MESP1 | NC_040860.1 | 14,845 | Instituto Tecnologico Vale, Brazil | 25-Feb-19 |
| 34 | Araneomorphae | Synspermiata | Pholcidae | *Mesabolivar* sp2. ITV1036I1 | MESP2 | NC_040859.1 | 14,941 | Instituto Tecnologico Vale, Brazil | 25-Feb-19 |
| 35 | Araneomorphae | Synspermiata | Dysderidae | *Parachtes romandiolae* isolate K352 | PASO | NC_044099.1 | 14,220 | IMEDEA, Spain | 11-Sep-19 |
| 36 | Araneomorphae | Synspermiata | Dysderidae | *Harpactocrates apennicola* isolate K350 | HAAP | NC_044081.1 | 14,213 | IMEDEA, Spain | 11-Sep-19 |
| 37 | Araneomorphae | Synspermiata | Pholcidae | *Pholcus phalangioides* | PHPH | NC_020324.1 | 14,459 | Universitaet Bonn, Germany | 07-Mar-13 |
| 38 | Mesothelae | Mesothelae | Liphistiidae | *Liphistius erawan* | LIER | NC_020323.1 | 14,197 | Universitaet Bonn, Germany | 07-Mar-13 |
| 39 | Mygalomorphae | Mygalomorphae | Dipluridae | *Phyxioschema suthepium* | PHSU | NC_020322.1 | 13,931 | Universitaet Bonn, Germany | 07-Mar-13 |
| 40 | Mesothelae | Mesothelae | Liphistiidae | *Heptathela hangzhouensis (Songthela hangzhouensis)* | SOHA | NC_005924.1 | 14,215 | Nanjing Normal University, China | 01-Feb-10 |
| 41 | Mygalomorphae | Mygalomorphae | Theraphosidae | *Ornithoctonus huwena* | ORHU | NC_005925.1 | 13,874 | Nanjing Normal University, China | 01-Feb-10 |
| 42 | Araneomorphae | Hypochilidae | Hypochilidae | *Hypochilus thorelli* | HYTH | NC_010777.1 | 13,991 | Portland State University, USA | 29-May-08 |
| 43 | Mygalomorphae | Mygalomorphae | Nemesiidae | *Calisoga_longitarsis* | CALO | EU523754.1 | 14,070 | Portland State University, USA | 16-Nov-12 |
| 44 | Araneomorphae | RTA clade | Miturgidae | *Cheiracanthium_triviale* | CHTR | MN334527.1 | 14,595 | Ministry of Environment & Forests, India | 29-Jan-20 |
| 45 | Araneomorphae | RTA clade | Oxyopidae | *Oxyopes_hupingensis* | OXHU | MK518391.1 | 15,078 | Guiyang College, China | 04-Mar-20 |

**Table S2** Primer list used to amplify uncertain fragments.

| **Primers** | **Direction** | **Sequence** |
| --- | --- | --- |
| tRNA region | F1 | TAGTTTTGATTTCTGGGCTTAT |
|  | R1 | ATTTCCATAGACCTACTTTC |
| D-loop region | F2 | TAAATCGTAAAATGGCGG |
|  | R2 | TCAAATAACCCATCTAACA |

**Fig. S1** Morphology and habitat of the intertidal spider, *Desis jiaxiangi.* A, E, D: female morphology; B, C: male left palp; D: vulva, dorsal view; F, Intertidal zone. Scale bars: A and E, 1 mm; B and C, 0.5 mm; D, 0.2 mm.
